# Serum Albumin as an Early Predictor of Proteinuria Recovery in Lupus Nephritis

**DOI:** 10.31138/mjr.040924.sai

**Published:** 2025-05-14

**Authors:** Fadi Kharouf, Taraneh Tofighi, Heather N Reich, Qixuan Li, Jiandong Su, Dafna D. Gladman, Zahi Touma

**Affiliations:** 1 University of Toronto Lupus Clinic, Division of Rheumatology, Schroeder Arthritis Institute, Krembil Research Institute, University Health Network, Toronto, ON, Canada;; 2 Department of Medicine, University of Toronto, Toronto, Canada;; 3 Division of Nephrology, University of Toronto, Toronto, Canada; 4 University of Toronto Lupus Clinic, Centre for Prognosis Studies in Rheumatic Diseases, Toronto Western Hospital, University Health Network, Toronto, ON, Canada

**Keywords:** systemic lupus erythematosus, lupus nephritis, serum albumin, proteinuria recovery

## Abstract

**Background/Purpose:**

Proteinuria, a slowly changing marker, is considered the best predictor of long-term renal outcomes in lupus nephritis (LN). In this study, we aimed to determine if serum albumin can serve as an early predictor of combined proteinuria recovery in LN.

**Methods:**

We studied patients diagnosed with LN with baseline and follow-up visits at 6–9 and 18–21 months. Receiver operating characteristic (ROC) curves were generated and the area under the curve (AUC) was analysed at different time points to test if serum albumin was a predictor of combined proteinuria recovery response (complete and partial proteinuria recovery, CPR+PPR) and primary efficacy proteinuria recovery (PEPR) at 6–9 and 18–21 months.

**Results:**

ROC curves for serum albumin level at baseline did not predict combined proteinuria recovery or PEPR at 6–9 or 18–21 months. However, serum albumin level at 6–9 months predicted combined proteinuria recovery at 6–9 months (AUC 0.77) and PEPR at 6–9 (AUC 0.83) and 18–21 months (AUC 0.83). Serum albumin absolute change (AUC=0.82) and percent change (AUC=0.81) from baseline to 6–9 months predicted the 6–9-month combined proteinuria recovery. Similarly, serum albumin absolute change (AUC=0.84) and percent change (AUC=0.82) from baseline to 18–21 months predicted the 18–21-month combined proteinuria recovery. A less pronounced, but similar signal was observed when PEPR was used as the endpoint at 6–9 (AUC 0.70 and 0.68, respectively) and 18–21 months (AUC 0.73 and 0.71, respectively).

**Conclusion:**

Serum albumin may serve as an accessible adjunct to proteinuria in assessing the clinical course and treatment response in LN.

## INTRODUCTION

Lupus nephritis (LN) is a manifestation of systemic lupus erythematosus (SLE) that accounts for significant morbidity and mortality, resulting in irreversible damage if not treated effectively.^1^ Unfortunately, nephritis is not a rare entity, affecting 35–60% of patients with SLE.^1–5^ Patients typically present with active lupus serology (low complement and positive anti-dsDNA antibodies), proteinuria, active sediment on urinalysis, and pathologic biopsy findings; renal function may also be impaired.^1,2,6^

The past few decades have witnessed the development of new therapeutics that show promise in achieving remission and preventing new flares.^7^ However, up to 20% of patients with LN still go on to develop end-stage kidney disease (ESKD) within the first decade of diagnosis.^8–11^ Furthermore, LN flares are frequent, with up to 66% of patients in remission experiencing them in follow-up.^12^ Repeated renal flares are associated with lower response to therapy and adverse prognosis in terms of kidney function and patient survival.^13^ Similarly, failure to achieve complete proteinuria recovery (CPR), defined as resolution of proteinuria (<500 mg/day), is associated with adverse long-term outcomes.^14^ As such, it is imperative to have biomarkers and tools that predict early response in order to guide further therapeutic interventions and clinical monitoring.

Clinical trials and observational studies have identified several outcome measures and endpoints to predict long-term renal and non-renal outcomes (chronic kidney disease [CKD], ESKD, cardiovascular events, etc.). Most studies look at complete and partial remission endpoints, which are comprised of measures such as spot urine samples for protein-creatinine ratio (PCR), serum creatinine, and urinary sediment.^15–18^ Presently, proteinuria is the primary outcome in the majority of research studies and in the clinical setting. The Euro-Lupus Nephritis Trial (ELNT) and the MAINTAIN Nephritis Trial (MNT) have identified proteinuria at 12 months as the best available predictor of long-term renal outcomes in LN, with a cut-off of 0.8 and 0.7 g/day respectively;^19,20^ this was also replicated in observational studies.^21^ Indeed, the European Alliance of Associations for Rheumatology (EULAR) recommendations for the management of SLE identify proteinuria as a principal determinant of response,^7,22^ noting that in monitoring renal response, reduction in proteinuria to less than 0.8 g/day carries more weight than monitoring for residual hematuria.^19^ The 2019 Joint EULAR and European Renal Association– European Dialysis and Transplant Association (EULAR/ERA–EDTA) recommendations^23^ and the Kidney Disease: Improving Global Outcomes (KDIGO) 2024 practical guidelines^24^ for the management of LN provide therapeutic targets to be achieved at different time points during treatment; therapy should aim for at least a 50% reduction of proteinuria (partial clinical response) by 6 months and below 500–700 mg/day by 12 months (complete clinical response), emphasising that the latter may take longer in patients with nephrotic-range proteinuria at baseline. We have previously demonstrated that recovery of proteinuria, a marker of response to therapy, is slow.^6^ In our study of 212 patients, only 28% of the patients achieved CPR at one year compared to 52% at two years, despite earlier improvement of other parameters suggestive of disease activity. As such, serum albumin, a potentially more rapidly responsive biomarker, may serve as an accessible and cost-effective adjunct to proteinuria in assessing early treatment response. We performed an analysis on a cohort of SLE patients with LN to determine if serum albumin can serve as an early predictor of combined proteinuria recovery (complete proteinuria recovery [CPR] + partial proteinuria recovery [PPR]) and primary efficacy proteinuria recovery (PEPR) at 6–9 and 18–21 months in LN patients receiving standard treatment.

## MATERIALS AND METHODS

### Study Cohort

Patients were selected from the prospective longitudinal Toronto Lupus cohort followed from 1990 to March 2020. All patients met the EULAR/American College of Rheumatology (ACR) 2019 classification criteria for SLE.^25^ Collection, storage, and use of clinical and laboratory data were conducted in accordance with the Declaration of Helsinki and approved by the Research Ethics Board of the University Health Network (REB#11-0397), Toronto, Canada. Signed informed consent was obtained from all patients.

### Inclusion criteria

LN definition: One-time 24-hour urine protein ≥0.5 g/day OR a urinary PCR ≥0.05 g/mmol OR biopsy-proven LN (class III, IV, V and III/IV+V). We mandated the above definitions to be attributed to SLE and associated with treatment initiated for the management of LN: new start/escalation with prednisone minimum of 10 mg/day total dose or dose escalation of 5 mg/day if already on prednisone or pulse glucocorticoids (methylprednisolone) or new immunosuppressive therapy. Patients were included if they had a baseline visit at the diagnosis of LN and subsequent follow-up visits at 6–9 months and preferably 18–21 months following diagnosis with data on serum albumin and proteinuria at all visits.

### Exclusion criteria

Patients with ESKD (dialysis or renal transplant or estimated glomerular filtration rate [eGFR] <15 mL/min/1.73m^2^) before the LN diagnosis date, patients with baseline serum albumin ≥ 40 g/L, and patients who were not followed long enough for the 6–9--month assessment.

### Patient Assessment

As is standard of care at the Toronto Lupus clinic, patients were seen at time intervals of two to six months regardless of disease activity, and more often if required. Patient assessments were based on a specified protocol and were comprised of history, physical examination, serological evaluation, and details on medications. For this study, we extracted laboratory results on serum creatinine, serum albumin, 24-hour proteinuria, spot urinary PCR, and kidney biopsy results, if available. With regards to the 24-hour proteinuria, samples were obtained from patients instructed to void in the morning and discard the urine, collecting from that point onwards over the next 24 hours. Samples were handled by the laboratory and appropriate measures were taken for standardised storage and preservation of samples.

### Study Design

Baseline was defined as the time of LN diagnosis (not necessarily the first LN episode in the disease course) per the aforementioned criteria. Serum albumin and urine PCR were followed for each patient at the 6–9 and 18–21-month points following the diagnosis of LN.

### Study Endpoints Definitions

The study endpoints included Combined Proteinuria Recovery (CPR+PPR) and Primary Efficacy Proteinuria Recovery (PEPR) (term derived from the proteinuric descriptor in the primary efficacy renal response endpoint used in the BLISS-LN trial).^26^ With consideration of Rovin [LUNAR] criteria,^27^ CPR was defined as PCR <0.5 g/g, and PPR was defined as a ≥50% decrease of proteinuria to PCR <1.0 g/g (if baseline PCR ≤3.0 g/g) or to PCR≤ 3.0 g/g (if baseline PCR >3.0 g/g). PEPR was defined as PCR **≤**0.7 g/g. We compared these outcomes to the SLE Disease Activity Index-2000 (SLEDAI-2K),^28^ which defines CPR as proteinuria <0.5g/24 hours, and the SLEDAI-2K Responder Index-50 (SLEDAI-2K RI-50),^29^ which defines PPR as a decrease of ≥50% in the level of proteinuria from baseline. Albumin recovery was considered complete at levels ≥35 g/L from a baseline of <30 g/L, and partial when ≥30 g/L but <35 g/L from a baseline of <30 g/L.

### Study Analysis

Descriptive statistics were employed to summarise patient characteristics. Unpaired t-test was conducted to compare continuous measurements, while the Chi-Square test was used for binary variables. Logistic regression was utilised to generate receiver operating characteristic (ROC) curves, assessing serum albumin as a predictor of combined proteinuria recovery and PEPR responses at 6–9 and 18–21 months. The area under the curve (AUC) was analysed for three predictors: (a) serum albumin level at baseline and 6–9 months (b) absolute albumin change from baseline to 6–9 and 18–21 months and from 6–9 months to 18–21 months, and (c) percent change from baseline to 6–9 and 18–21 months and from 6–9 to 18–21 months. AUC values of 1.0–0.91 indicate outstanding performance, 0.81–0.90 excellent, 0.71–0.8 good, 0.61–0.7 fair, and ≤0.6 poor performance.^30^

The results were presented as AUC, with odds ratio (OR) and 95% confidence interval (CI). Optimal cutoff points of serum albumin in g/L, as well as positive and negative predictive values (PPV and NPV) were reported. Notably, Bonferroni corrections were applied to the confidence intervals of the AUCs, with a significant level of 0.004. We hypothesised that both absolute and percent change of serum albumin (baseline to 6–9 months), but not baseline serum albumin level, will predict proteinuria endpoints at 6–9 months, and to a lesser degree at 18–21 months (because of the limited sample size). We also expected that both absolute and percent change of serum albumin (baseline to 18–21 months) would predict proteinuria endpoints at 18–21 months. The percentage of patients achieving combined CPR+PPR is greater than the percentage of patients achieving PEPR, thus the AUC results were expected to perform better with the former.

## RESULTS

### Patient Characteristics

In total, 112 patients (85.7% female) with a baseline visit (time of diagnosis of LN) and a 6–9-month follow-up visit were included in the study (**Table 1**). The mean ± standard deviation (SD) age at LN diagnosis was 34.8 ± 12.1 years, with a mean disease duration of 4.9 ± 5.3 years. Ninety-eight percent of the patients received glucocorticoids at the time of LN diagnosis at a mean glucocorticoid dose of 27.1 ± 13.8 mg/day. The majority of patients were also treated with antimalarials (61.8%) and about half (50.5%) received immunosuppressives at baseline; the latter increased to 93 (83.0%) at 6–9 months. The mean SLEDAI-2K was 15.6 ± 7.2, with a mean SLICC/ACR Disease Damage Index (SDI) of 0.4 ± 0.9 at baseline. Of the 81 patients (72.3%) who had a renal biopsy, the majority were World Health Organisation (WHO)/International Society of Nephrology (ISN) class III or IV (pure proliferative disease) (*n*=39), followed by mixed III/IV+V biopsies (*n*=22), and class V LN (*n*=20). Of this cohort of 112 patients, 68 (60.7%) had an additional 18–21-month visit after baseline.

**Table 1.** Baseline characteristics of a cohort of 112 lupus nephritis patients.

Female	96 (85.7%)
Age at LN (mean years ± SD)	34.8 ± 12.1
Disease duration at LN (mean years ± SD)	4.9 ± 5.3
Biopsy class by ISN/WHO criteria (N = 81)	
III	10 (12.3%)
IV	29 (35.8%)
V	20 (24.7%)
IV/III +V	22 (27.2%)
Race	
Black	26 (23.2%)
White	50 (44.6%)
Chinese	18 (16.1%)
Others	18 (16.1%)
Serum albumin at LN (g/L, mean ± SD)	32.4 ± 6.4 (n=112)
24-hour proteinuria at LN (gram/day, mean ± SD; median	
interquartile range] )	2.6 ± 1.7; 2.3 [1.3, 3.6]
Serum creatinine at LN diagnosis (umol/L, mean ± SD)	82.9 ± 48.5 (n=112)
SLEDAI-2K score at LN diagnosis (mean ± SD)	15.6 ± 7.2
SDI score (mean ± SD)	0.4 ± 0.9
Systolic blood pressure at diagnosis (mmHg, mean ± SD)	125.0 ±19.8
Diastolic blood pressure at LN diagnosis	76.4 ± 12.5
(mmHg, mean ± SD)	
Treated with ACEi or ARB at LN diagnosis	38 (33.9%)
Treated with glucocorticoids at LN diagnosis	110 (98.2%)
Prednisone dose (mg/day) at LN (mean ± SD) diagnosis	27.1 ± 13.8
Treated with antimalarials at LN diagnosis	68 (61.8%)
Treated with immunosuppressives at LN diagnosis*	54 (50.5%)

ACEi: angiotensin-converting enzyme inhibitor; ARB: angiotensin receptor blocker; ISN: International Society of Nephrology; LN: lupus nephritis; SD: standard deviation; SLEDAI-2K: systemic lupus erythematosus disease activity index-2000; WHO: World Health Organisation.

* At 6–9 months, 93 (83%) patients received immunosuppressives. The most commonly used medications were mycophenolate mofetil, azathioprine, and cyclophosphamide, in that order. Two patients were treated with calcineurin inhibitors (cyclosporine or tacrolimus).

### Laboratory Results

The mean serum albumin amongst the 112 patients was 32.4 g/L ± 6.4 at baseline, 36.7 g/L ± 7.1 at the 6–9-month visit, and 38.9 g/L ± 6.9 (n=68) at the 18–21-month visit. The mean 24-hour proteinuria was 2.6 g/day ± 1.7 at baseline, 1.6 g/day ± 1.7 at the 6–9-month visit, and 1.3 g/day ± 1.1 at the 18–21-month visit.

### Proteinuria Recovery

More than half of the patients demonstrated a combined proteinuria recovery at 6–9 months (n=58; 51.8%). Sixty percent achieved this outcome at 18–21 months (n=41; 60.3%). The results were similar using both Rovin [LUNAR] and SLEDAI-2K-based endpoints. After excluding patients with baseline proteinuria ≤0.7 g/day, 38% (36 out of 95) achieved PEPR at 6–9 months; 59% (35 out of 59) did so at 18–21 months (**Table 2**).

**Table 2.** Combined (complete and partial) proteinuria recovery, as well as primary efficacy proteinuria recovery at the 6–9 and 18–21-month visit.

	**6–9-month visit (n=112)**		**18–21-month visit (n=68)**	
	Rovin [LUNAR]	SLEDAI-2K or SLEDAI-2K RI50	Rovin [LUNAR]	SLEDAI-2K or SLEDAI-2K RI50
**CPR and PPR**	58 (51.8%)	62 (55.4%)	41 (60.3%)	43 (63.2%)
**PPR**	24 (21.4%)	28 (25.0%)	7 (10.3%)	9 (13.2%)
**CPR**	34 (30.4%)	34 (30.4%)	34 (50.0%)	34 (50.0%)
	6–9-month visit (n=95* )		18–21-month visit (n=59* )	
**PEPR**	36 (37.8%)	---	35 (59.3%)	---

CPR: complete proteinuria recovery; PEPR: primary efficacy proteinuria recovery; PPR: partial proteinuria recovery; SLEDAI-2K: systemic lupus erythematosus disease activity index-2000; SLEDAI-2K RI50: SLEDAI-2K responder index-50.

* Patients with baseline proteinuria ≤0.7 g/day were excluded from the analysis.

There was a statistically significant difference in the mean 6–9-month serum albumin between patients who achieved combined proteinuria recovery at 6–9 months and those who did not; 39.9 g/L ± 5.1 (n=58) vs. 33.2 g/L ± 7.3 (n=54), respectively, p≤0.001. There was no significant difference between the two groups in terms of baseline serum albumin levels (32.3 g/L ± 6.7 (n=58) vs. 32.4 g/L ± 6.2 (n=54), respectively, p=0.96). Mean serum creatinine at the 6–9-month visit was 73.1 μmol/L ± 27.6 (n=58) in patients who achieved combined proteinuria recovery compared to 112.6 μmol/L ± 113.4 (n=54) in those who did not (p=0.01). Baseline serum creatinine was lower in the former group, but this did not meet statistical significance (74.6 μmol/L ± 31.8 (n=58) vs. 91.7 μmol/L ± 60.7 (n=54), p=0.06).

### Serum Albumin as a Predictor of Proteinuria Recovery Endpoints

#### Absolute albumin level as a predictor of response at 6–9 and 18–21 months endpoints:

Absolute albumin level at 6–9 months predicted the 6–9-month combined proteinuria recovery (AUC=0.77, OR 1.20, 95% CI 0.65–0.90) and PEPR (AUC=0.83, OR 1.28, 95% CI 0.71–0.95), as well as the 18–21-month PEPR (AUC=0.83, OR 1.27, 95% CI 0.66–1.00). All other absolute albumin levels were not predictive of the different outcomes at 6–9 and 18–21 months **(Table 3)**.

**Table 3.** Performance of different variables to predict combined proteinuria recovery (CPR+PPR) and PEPR endpoints.

**Variables**	**AUC, OR** **95% CI**	**PPV, NPV, OC**	**AUC, OR** **95% CI**	**PPV, NPV, OC**
	**CPR+PPR at 6–9 months**		**CPR+PPR at 18–21 months**	
Absolute albumin at baseline	AUC=0.41, OR=0.95 95% CI 0.25–0.56	PPV=0.25, NPV=0.60, OC=19.50	AUC=0.53, OR 1.00 95% CI 0.37–0.70	PPV=0.42, NPV=0.68, OC=34.50
Absolute albumin at 6–9 months	AUC 0.77, OR 1.20 95% CI 0.65–0.90	PPV=0.71, NPV=0.69, OC=37.50	AUC=0.67, OR 1.08 95% CI 0.52–0.82	PPV=0.52, NPV=0.82, OC=36.50
Absolute change from baseline to 6–9 months	AUC=0.82, OR 1.22 95% CI 0.70 −0.93	PPV=0.75, NPV=0.78, OC=3.50	AUC=0.62, OR 1.06 95% CI 0.47–0.78	PPV=0.47, NPV=0.81, OC=2.50
Percent change from baseline to 6–9 months	AUC=0.81, OR 1.04 95% CI 0.69 - 0.93	PPV=0.77, NPV=0.77, OC=10.96	AUC=0.62, OR 1.01 95% CI 0.47–0.77	PPV=0.47, NPV=0.81, OC=7.90
Absolute change from baseline to 18–21 months	NA	NA	AUC=0.84, OR 1.25 95% CI 0.69–1.00	PPV=0.62, NPV=0.96, OC=2.00
Percent change from baseline to 18–21 months	NA	NA	AUC=0.82, OR 1.05 95% CI 0.62–0.96	PPV=0.62, NPV=0.96, OC=5.13
Absolute change from 6–9 to 18–21 months	NA	NA	AUC=0.66, OR 1.13 95% CI 0.45–0.87	PPV=0.48, NPV=0.94, OC=−1.50
Percent change from 6–9 to 18–21 months	NA	NA	AUC=0.63, OR 1.03 95% CI 0.40–0.85	PPV=0.50, NPV=0.94, OC=−2.38
	**PEPR at 6–9 months**		**PEPR at 18–21 months**	
Absolute albumin at baseline	AUC=0.59, OR 1.06 95% CI 0.42–0.76	PPV=0.44, NPV=0.76, OC=28.50	AUC=0.65, OR 1.06 95% CI 0.45–0.86	PPV=0.75, NPV=0.55, OC=34.50
Absolute albumin at 6–9 months	AUC=0.83, OR 1.28 95% CI 0.71–0.95	PPV=0.66, NPV=0.83, OC=38.50	AUC=0.83, OR 1.27 95% CI 0.66–1.00	PPV=088, NPV=0.74, OC=36.5
Absolute change from baseline to 6–9 months	AUC=0.70, OR 1.10 95% CI 0.55–0.85	PPV=0.53, NPV=0.83, OC=3.50	AUC=0.73, OR 1.12 95% CI 0.52–0.94	PPV=0.75, NPV=0.74, OC=1.00
Percent change from baseline to 6–9 months	AUC=0.68, OR 1.01 95% CI 0.53–0.84	PPV=0.53, NPV=0.83, OC=10.13	AUC=0.71, OR 1.02 95% CI 0.50–0.93	PPV=0.75, NPV=0.74, OC=2.56
Absolute change from baseline to 18–21 months	NA	NA	AUC=0.72, OR 1.10 95% CI 0.45–0.90	PPV=0.81, NPV=0.90, OC=2.00
Percent change from baseline to 18–21 months	NA	NA	AUC=0.70, OR 1.01 95% CI 0.46–0.94	PPV=0.77, NPV=0.93, OC=1.28
Absolute change from 6–9 months to 18–21 months	NA	NA	AUC=0.56, OR 1.01 95% CI 0.32–0.81	PPV=0.69, NPV=0.82, OC=−1.5
Percent change from 6–9 months to 18–21 months	NA	NA	AUC=0.53, OR 0.99 95% CI 0.27–0.79	PPV=0.69, NPV=0.90, OC=−5.16

AUC: under the curve; OR: odds ratio; CI: confidence interval; PPV: positive predictive value; NPV: negative predictive value; OC: optimal cutoff (g/L); CPR: complete proteinuria recovery; PPR: partial proteinuria recovery; NA: not available; PEPR: primary efficacy proteinuria recovery.

Poor; AUC of ≤0.60

Fair; AUC of 0.61–0.70

Good; AUC of 0.71–0.80

Excellent; AUC of 0.81–0.90

Outstanding; 0.91–1.0

#### Baseline to 6–9 months as a predictor of the 6–9-month endpoints:

**Figure 1.**
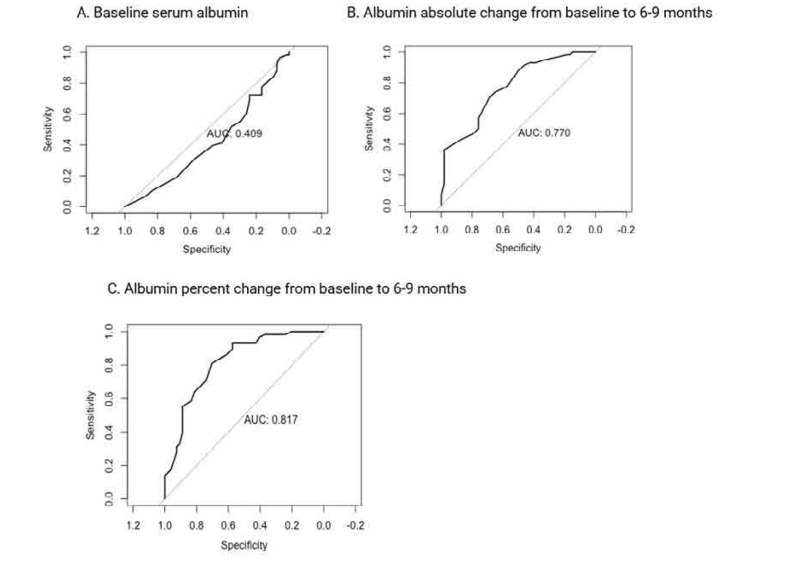
**(A–C)** ROC curve predicting 6–9-month combined proteinuria recovery endpoints (defined as PPR+CPR by Rovin criteria [LUNAR]) using absolute and percent change of serum albumin from baseline to 6–9 months (n= 112). ROC: receiver operating characteristic; PPR: partial proteinuria recovery; CPR: complete proteinuria recovery.

Serum albumin absolute change (AUC= 0.82, OR 1.22, 95% CI 0.70–0.93) and percent change (AUC=0.81, OR 1.04, 95% CI 0.69–0.93) from baseline to 6–9 months predicted the 6–9-month combined proteinuria recovery (**Table 3** and **Figure 1**). Although to a lower extent, serum albumin absolute change (AUC=0.70, OR 1.10, 95% CI 0.55–0.85) and percent change (AUC=0.68, OR 1.01, 95% CI 0.53–0.84) from baseline to 6–9 months also predicted the 6–9-month PEPR.

#### Baseline to 6–9 months as a predictor of the 18–21-month endpoints:

**Figure 2.**
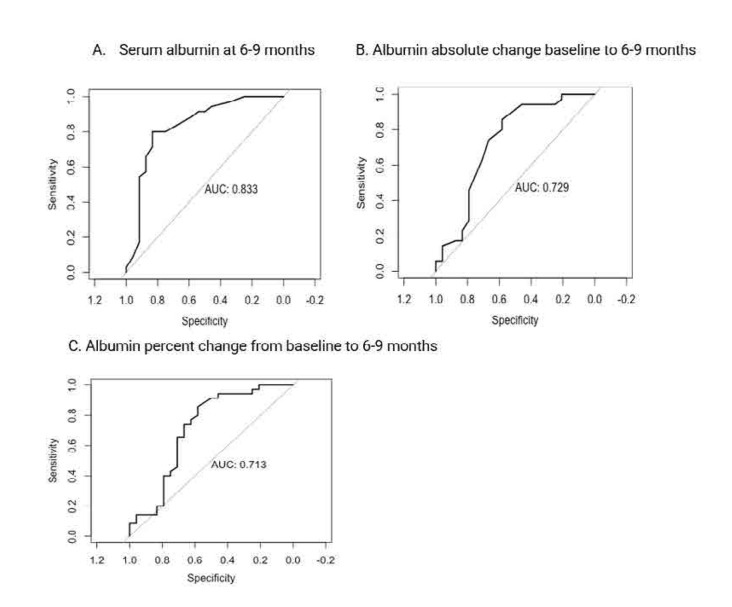
**(A–C)** ROC curve predicting 18–21-month PEPR (defined as proteinuria ≤0.7 g/day) using absolute and percent change of serum albumin from baseline to 6–9 months (n=59). ROC: receiver operating characteristic; PEPR: primary efficacy proteinuria recovery.

Neither serum albumin absolute change (AUC=0.62, OR 1.06, 95% CI 0.47–0.78) nor percent change (AUC=0.62, OR 1.01 CI 0.47–0.77) from baseline to 6–9 months predicted the 18–21-month combined proteinuria recovery. However, when PEPR was used as the outcome, absolute change (AUC=0.73, OR 1.12, 95% CI 0.52–0.94) and percent change (AUC=0.71, OR 1.02, 95% CI 0.50–0.93) from baseline to 6–9 months were both predictive (**Figure 2**).

#### Baseline to 18–21 months as a predictor of the 18–21-month endpoints:

**Figure 3.**
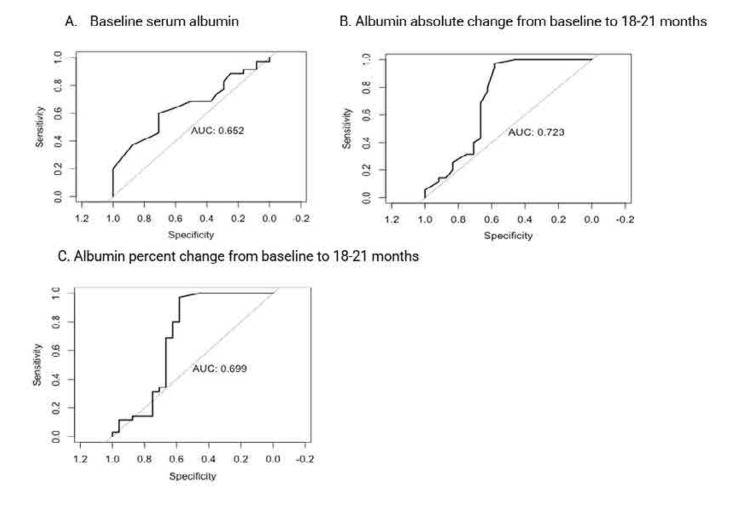
(A–C) ROC curve predicting 18–21-month combined proteinuria recovery (defined as PPR+CPR by Rovin criteria [LUNAR]) using absolute and percent change of serum albumin from baseline to 18–21 months (n= 68). ROC: receiver operating characteristic; PPR: partial proteinuria recovery; CPR: complete proteinuria recovery.

Serum albumin absolute change (AUC=0.84, OR 1.25, 95% CI 0.69–1.00) and percent change (AUC=0.82, OR 1.02, 95% CI 0.62–0.96) from baseline to 18–21 months predicted the 18–21-month combined proteinuria recovery (**Figure 3**). Although to a lower extent, serum albumin absolute change (AUC=0.72, OR 1.10, 95% CI 0.45–0.90) and percent change (AUC=0.70, OR 1.01, 95% CI 0.46–0.94) from baseline to 18–21 months also predicted the 18–21-month PEPR.

#### 6–9 months to 18–21 months as a predictor of the 18–21-month endpoints:

Neither serum albumin absolute change nor percent change from 6–9 months to 18–21 months predicted combined proteinuria recovery or PEPR **(Table 3)**.

PPV, NPV, and optimal cutoffs of serum albumin (in g/L) are also shown in **Table 3**.

## DISCUSSION

Our study demonstrates that changes in serum albumin are predictive of proteinuria recovery endpoints in LN. We found serum albumin absolute and percent change from baseline to 6–9 months and from baseline to 18–21 months to be predictive of combined proteinuria recovery, and to a lesser extent, of PEPR at 6–9 and 18–21 months, respectively. The better results obtained with combined proteinuria recovery compared to PEPR are most likely explained by the higher number of patients meeting this outcome at the two time points, as shown in **Table 2**.

We also found serum albumin absolute and percent change from baseline to 6–9 months to predict the 18–21 month PERP. The better prediction of PERP as compared to combined proteinuria recovery in this case is likely related to the relatively lower proteinuria levels of PERP achievers at 18–21 months (0.39 g/day ± 0.27 vs. 0.43 g/day ±0.28). While serum albumin level at baseline was not predictive of combined proteinuria recovery or PEPR at 6–9 or 18–21 months, its level at 6–9 months predicted the 6–9-month combined proteinuria recovery and PERP, as well as the 18–21-month PEPR. These findings may be attributed to the higher levels of serum albumin achieved with time (32.4 g/L ± 6.4 at baseline vs. 36.7 g/L± 7.1 at 6–9 months), which better correlate with proteinuria recovery endpoints.

We also demonstrated a statistically significant difference in the mean serum albumin at 6–9 months between patients who achieved combined proteinuria recovery at 6–9 months and those who did not (39.9 g/L as compared to 33.2 g/L, respectively, p≤0.001), which was not observed with other disease markers, such as complement and anti-dsDNA. This differentiation between the two groups based on serum albumin adds value to its use as an indicator of response. Furthermore, the gradual decrease in the proteinuria level from baseline to 6–9 to 18–21 months was paralleled by a gradual increase in serum albumin levels, with more change in the latter occurring from baseline to 6–9 months.

Our results are remarkable as they prove a useful role for an easily accessible blood test in predicting response to therapy. While urinary PCR is a feasible test to do, proteinuria is known to be a slowly changing marker, often taking years to resolve.^6^ Additionally, in LN cases presenting with nephrotic-range proteinuria, in which recovery may take longer to achieve,^23^ changes in serum albumin from baseline to 6–9 months (and the absolute serum albumin level at that time), would provide a valuable tool for predicting a later response, i.e. PEPR at 18–21 months. Thus, by having a test that predicts the inherently slow proteinuric response, appropriate decision-making on management can be facilitated. In this regard, the advantage of serum albumin is that it is often included in routine bloodwork and represents a low-cost test.

We hypothesise that the predictive power of serum albumin can be explained by several mechanisms, including increased albumin catabolism in active SLE and the renal losses attributable to active LN. As such, absolute and percent change of serum albumin mirror those of proteinuria recovery as renal (and systemic) inflammation subsides throughout treatment.

This study adds to the body of literature exploring the role of serum albumin in LN. One of the early studies by Yip et al.^31^ demonstrated an association between the SLEDAI-2K score and serum albumin, strongest in SLE patients with LN compared to those without. The authors suggested that serum albumin may serve as a useful screening test in SLE, prompting the ordering of 24-hour proteinuria studies. Yuan et al.^32^ demonstrated a statistically significant difference in baseline albumin levels between patients with mild (pathological class I–II) and severe LN (pathological class III–V) and between patients with normal kidney function and those with reduced eGFR (<60 mL/min/1.73m^2^). Additionally, an earlier study by Sui et al.^33^ found serum albumin levels to be associated with the severity and activity of renal damage in LN patients with nephrotic syndrome; higher levels of proteinuria and lower levels of serum albumin were positively correlated with activity and chronicity indices. Of note, Domingues et al.^34^ showed that a serum albumin cut-off of 37 g/L one year after renal biopsy was predictive of 48-month favourable long-term renal outcomes (defined as the absence of doubling of serum creatinine, creatinine >4 mg/dL if initial >2.5 mg/dL, and ESKD), with a sensitivity of 94% and specificity of 87%.

Limitations of our study include single-centre design, although our patient cohort represents a demographically diverse population, given our hospital serves as a specialised referral centre within the country. Additionally, we did not account for other aetiologies influencing albumin levels, such as gastrointestinal loss, advanced liver disease, and severe malnutrition. These, however, remain uncommon in our patient population. Moreover, we used urinary PCR and 24-hour proteinuria interchangeably in our definition of proteinuria. Although small discrepancies can exist between both,^35,36^ urinary PCR has been validated as a measure of proteinuria in LN.^36,37^ Finally, we captured follow-up data on the 60.7% of subjects that had available visits and lab values at 18–21 months. In fact, the choice of the 6–9 and 18–21 point marks was directed by the availability of data, otherwise, assessments at earlier and later times would have been feasible.

In summary, our analysis is among the first studies in literature to demonstrate the role of serum albumin levels and their longitudinal change, in predicting the recovery of proteinuria as a measure of response to therapy. As serum albumin is easily accessible, cost-effective, and often included in routine bloodwork, it may serve as a useful clinical adjunct to proteinuria in clinical and research settings. This may have implications for future treat to target guidelines in LN.

## CONTRIBUTORSHIP

All authors contributed to the drafting or revising of the article for important intellectual content. All authors approved the final version of the article to be published.

Study conception and design: FK, TT, HNR, JS, QL, DDG, ZT

Acquisition of data: DDG, ZT

Analysis and interpretation of data: FK, TT, HNR, JS, QL, DDG, ZT

## DATA SHARING STATEMENT

Dr. Touma had full access to all of the data in the study and takes responsibility for the integrity of the data and the accuracy of the data analysis.

All authors were involved in the study conception and design, acquisition of data, analysis, and interpretation of data.

## DISCLOSURES & CONFLICT OF INTEREST

No disclosures for all authors. The authors declare no conflict of interest.

## FUNDING ACKNOWLEDGEMENT

No funding was received for this manuscript.

The Toronto Lupus Program is supported by Lupus Ontario, the Schroeder Arthritis Institute and donations from the Kathi and Peter Kaiser family and the Lou and Marissa Rocca family. Dr Zahi Touma is supported by the University of Toronto Department of Medicine.

## ETHICAL APPROVAL INFORMATION

This research study was approved by the University Health Network Research Ethics Board (REB Study#: 11-0397). All individuals have provided written informed consent for this research study.
